# Diagnostic delay in soft tissue tumors: a single-center study of a university cancer center with a focus on health services research

**DOI:** 10.1186/s12913-022-07891-w

**Published:** 2022-04-06

**Authors:** Tobias M. Ballhause, Alonja Reiter, Alexander Korthaus, Karl-Heinz Frosch, Carsten W. Schlickewei, Matthias H. Priemel

**Affiliations:** 1 Department of Trauma and Orthopedic Surgery, University Medical Center Hamburg-Eppendorf, Martinistr. 52, Hamburg, Germany; 2 Department of Trauma Surgery, Orthopedics and Sports Traumatology, BG Hospital Hamburg, Hamburg, Germany

**Keywords:** Soft tissue tumors, Sarcoma, Cancer, Sarcoma center, MRI

## Abstract

**Background:**

Lumps and soft tissue tumors (STT) are frequent reasons for consulting a physician. Most STT are benign, and lumps are not always associated with a tumor. MRI is the most advanced imaging modality to assist a provisional diagnosis of STT. Only a small fraction of STT is malignant, these soft tissue sarcomas are known for their aggressive growth.

The study aims to analyze the influence of the MRI report on the speed of treatment of patients with suspected STT.

**Methods:**

This was a retrospective, longitudinal, single-center study from 2011–2020. We included adult patients who had biopsies or resections of masses suspicious for STT in MRI exams. MRI reports were classified as benign (I), intermediate/unclear (II), or malignant (III). For these cohorts, time was statistically analyzed from MRI scan to first contact with the University cancer center (UCC) and surgery. Furthermore, distance in kilometers from the patients´ home to the UCC was examined and compared to age and suspected malignancy.

**Results:**

Three hundred two patients (♀130; ♂172) were included. Histologic analyses revealed 286 tumors. The average age of the patients was 54.7(SD: 16.2) years. Malignant tumors were more often suspected in older patients (*p* = 0.0098). Patients with a benign diagnosed tumor in MRI contacted the UCC after an average of 31.3 (SD: 47.8) days. In contrast, patients with suspicion of a malignant tumor contacted the UCC significantly earlier, after 14.1 days (SD: 17.1); *p* = 0.0098. Likewise, the time between first contact and biopsy/resection was 32.8 days (SD: 35.7) for suspiciously benign tumors, and potentially malignant tumors were treated significantly faster 14.8 (SD: 16.0) days; (*p* = 0.028). Patients traveled on average 47.5 km (range: 0.5–483) to contact a specialized physician at the UCC. Suspected degree of malignancy or patient´s age had no statistical influence on traveled distance.

**Discussion:**

The treatment speed depended to a great extent on the suspected malignancy of the STT in the MRI report. The provisional diagnoses from the radiologist highly influenced the time delay between MRI scan and first contact to the UCC and surgical treatment. No discrimination of age or distance to the UCC was observed in this study.

**Supplementary Information:**

The online version contains supplementary material available at 10.1186/s12913-022-07891-w.

## Background

Sarcomas of the soft tissue are fairly rare tumors with an incidence of 4.7/100,000 [1]. More than 70 subtypes of sarcomas are known, and their different presentations make a distinction with benign growths even more difficult [2]. However, doctors are frequently contacted by patients with soft tissue swellings. After clinical assessment, magnetic resonance imaging (MRI) is the most effective diagnostic tool for soft tissue imaging [3, 4]. Radiologists are rarely confronted with sarcomas of the soft tissue due to the low incidence [5]. This makes further differentiation of a soft tissue tumor (STT) in a benign or malignant tumor, even more, difficult [6]. Sarcomas of the soft tissue collectively account for only 1% of all malignant tumors [7]. They can be highly malignant tumors with overall 5-year survival of 50–60% [8]. Early diagnosis of the sarcoma increases survival chances and reduces the magnitude of surgery [9, 10].

Evidence exists that treatment in a specialized sarcoma center improves treatment and consequently increases survival [11, 12]. National guidelines have led to recommendations for the treatment of sarcoma patients [13, 14]. The literature contains reports on delays of diagnostics on sarcomas, and there are multiple differences among different health care systems in various countries [15, 16].

This study aimed to depict the trajectory of patients with suspected soft tissue tumors in MRI from a university cancer center (UCC) in a metropolitan area in Western Europe with a special focus on health services and the timing of diagnosis.

## Methods

**Fig. 1:**
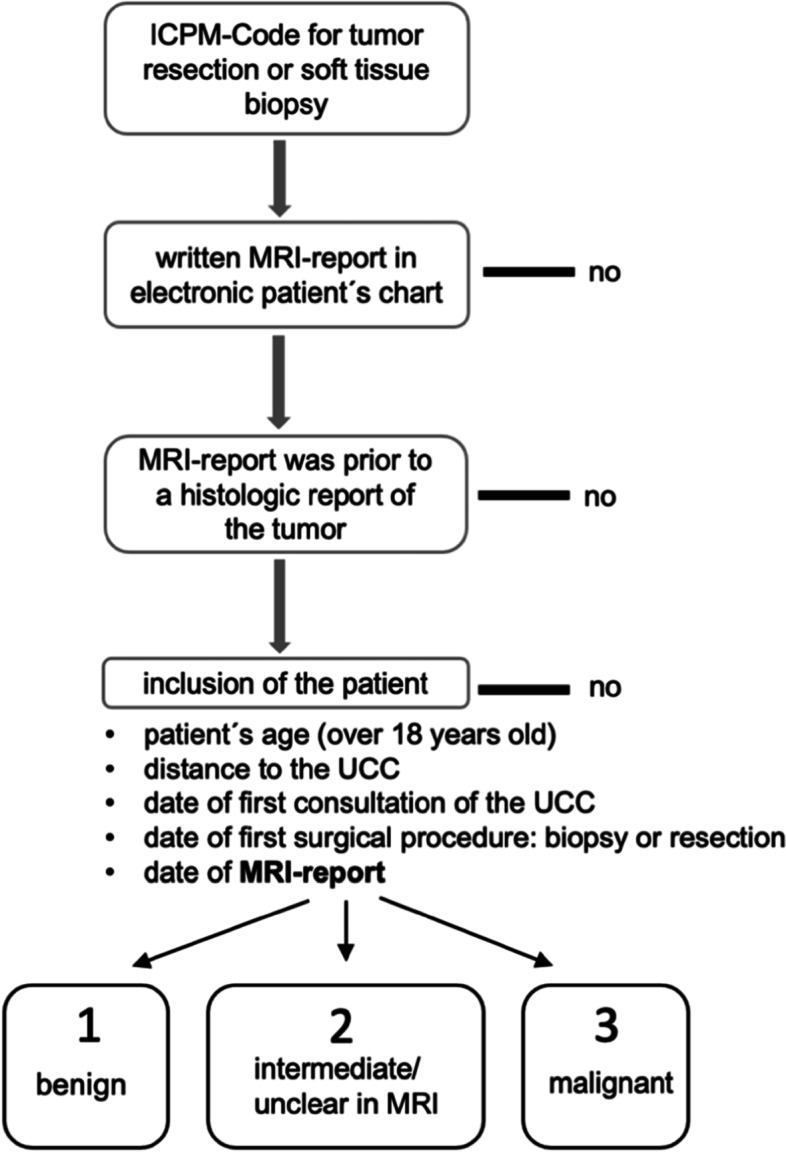
Inclusion criteria of the study. Patients were initially selected with help of the German version (OPS-301) of the ICPM (International Classification of Procedures in Medicine). Patients were classified into three groups according to the suspected malignancy of the tumor in the MRI-report´s provisional diagnosis

The study has a retrospective, longitudinal, single-center design. Institutional Review Board (Ethikkommission der Hamburger Ärztekammer) approval was given (WF-071/20). Patients were analyzed from 2011 to 2020 in a tertiary academic hospital, which is a regional reference center of sarcoma in a Western European metropolitan area. All patients had received a biopsy or resection of a suspected STT in our institution. The inclusion criteria were age over 18 years, MRI-based diagnosis of a tumor with written MRI report in the electronic patients´ chart, surgical treatment in our institution, and written report of the histologic analysis (Fig. 1). The exclusion criteria were MRI diagnosis made after the histologic diagnosis and no biopsy/resection or missing histopathologic analysis of the tumor. Most MRIs were conducted by external radiologists (86%). 14% were conducted at our institution. All MRI-reports were written before the histopathological result of the tumor was known. Only 17 patients had their MRI diagnosis performed after first contact with the UCC and were not included in this statistical analysis. Most of these patients only had sonographic assessments of the mass at the time of the first contact.

We first hypothesized that patients with a suspicion of a malignant soft tissue tumor would receive priority treatment. Therefore, the radiologic diagnosis was classified as benign (I), intermediate/unclear (II), or malign (III). In some cases, the radiologist described the tumor as “unclear, biopsy is recommended”; these were categorized as intermediate/unclear (II). The time frame was analyzed from MRI diagnosis to consultation of the UCC and surgical treatment.

Secondly, we expected patients with an MRI report of a malignant tumor to travel further to meet a specialist. The distance from the registered address of the patient to the UCC was measured (in kilometers) with Google Maps (Google LCC, Mountain View, CA).

The statistical analysis was performed using GraphPad Software 9 (Los Angeles, CA, USA). Parametric distribution was tested with the Shapiro–Wilk test. Non-parametrically distributed data were analyzed via the Kruskal–Wallis test. Parametrically distributed data were assessed with two-way ANOVA using Tukey´s multiple comparisons test. Correlation was examined with Spearman rank test for non-parametric data and Pearson´s test for parametrically distributed data. A confidence interval of 95% was chosen for all tests.

## Results

There were 302 lumps in 302 patients that met the inclusion criteria (130 women and 172 men). Histologic analysis revealed that not all MRI-examined masses were cancerous; 16 of the 302 lumps had no tumor tissue. According to the provisional diagnosis in MRI belonged 99 masses to category I (benign), 48 to category II (intermediate or unclear), and 155 masses were categorized as potentially malignant (III). The final histopathologic results are listed in detail in Table 1. The concordance of provisional MRI-based diagnosis to the final histopathologic result was 67.5%.

**Table 1:** Histological results of 302 in MRI suspected soft tissue tumors

Entity	n	Subtype (n)
Benign tumors	lipoma	116
*n* = 153	fibrolipoma	4
	lipoma abroscens	1
	spindle cell lipoma	2
	angiolipoma	6
	hibernoma	2
	ganglion	2
	myxoma	13
	fibroma	5
	desmoid tumor	1
	tenosynovial giant cell tumor	1
Intermediate tumors	ALT	39
*n* = 46	schwannoma	4
	desmoid fibromatosis	3
Malignant tumors	DDLPS	5
*n* = 87	NOSLPS	3
	MXLPS	32
	PMLPS	22
	chondrosarcoma	2
	fibrosarcoma	1
	spindle cell sarcoma	2
	synovial sarcoma	3
	rhabdomyosarcoma	4
	leiomyosarcoma	2
	round-cell sarcoma	3
	Ewing-like sarcoma	1
	myofibroblastoma	3
	lymphoma	2
	dermatofibrosarcoma protuberans	2
other	hemangioma	6
*n* = 16	hemorrhage	2
	fat necrosis	3
	cyst	3
	granulomatous inflammation	1
	myositis ossificans	1

**Fig. 2:**
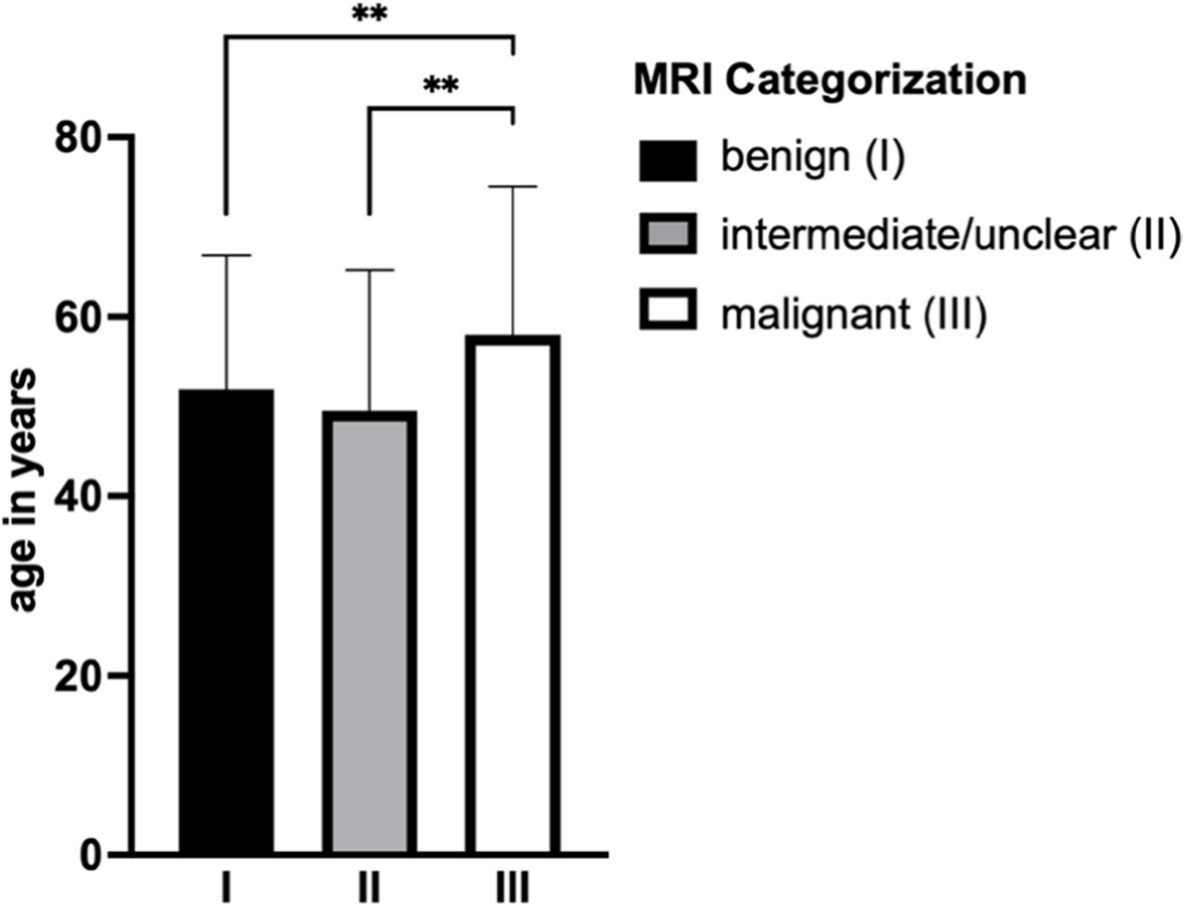
Age distribution of patients with soft tissue tumors diagnosed by MRI. Statistical significance is indicated by **p < 0.005

**Fig. 3:**
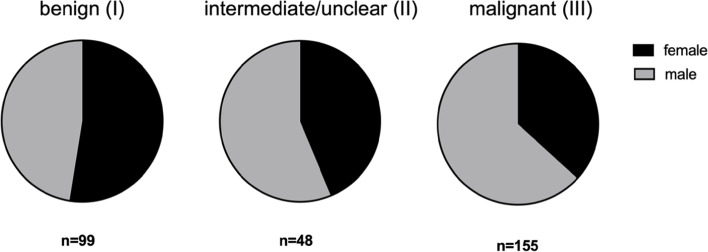
Sex distribution in the different cohorts according to the MRI-reported malignancy. Altogether, 132 women were included and 170 men

The average patient age was 54.7 years (range: 19–94 SD: 16.2). Patients with the diagnosis of a benign STT were on average 51.9 (SD: 14.9) years old. Intermediate/unclear tumors were suspected in patients with an average age of 49.6 (SD: 15.6) years. Malignant tumors were suspected in patients with an average age of 58.0 (SD: 16.5) years and these were significantly older in comparison to patients with benign (*p* = 0.0098) or intermediate/unclear tumors (*p* = 0.0041) (Fig. 2). More men than women were included in the study. But the distribution of tumors according to their suspected malignancy was not statistically related to gender (Fig. 3).

**Fig. 4:**
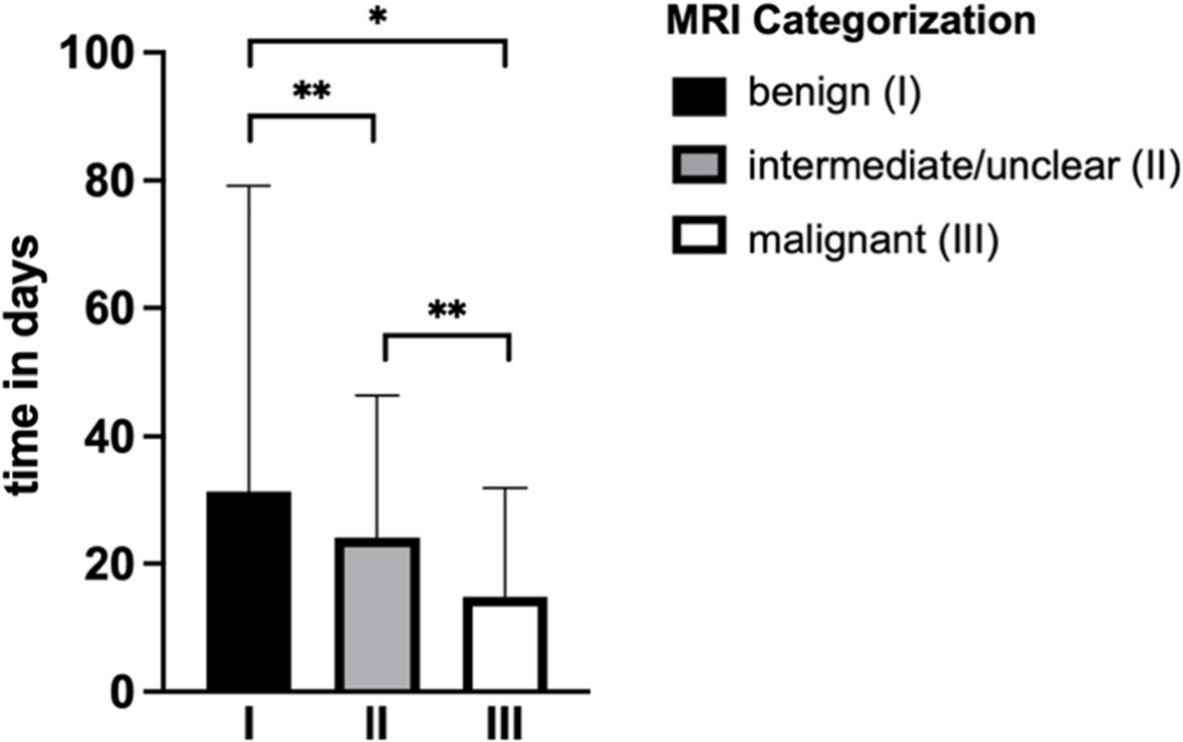
Time interval between diagnostic and consultation of a specialist. Statistical significance is indicated by *p < 0.05, **p < 0.005

On average 31.3 days (SD: 47.8) were between MRI and first contact to the UCC in patients with a suspected benign STT. The diagnostic time delay was significantly shortened in patients with intermediately/unclearly classified tumors in MRI-report to 24.1 days (SD: 22.3) (*p* < 0.0001). STT rated by the radiologists as malignant presented at the UCC after 14.8 days (SD: 17.1). Thus, the diagnostic delay was significantly shorter in comparison to benign (*p* = 0.028) and intermediately/unclearly rated tumors (*p* < 0.0001) (Fig. 4). No correlation was detected between a higher suspected degree of malignancy and a smaller time frame between MRI scan to first contact to the UCC (*r* = -0.1338).

**Fig. 5:**
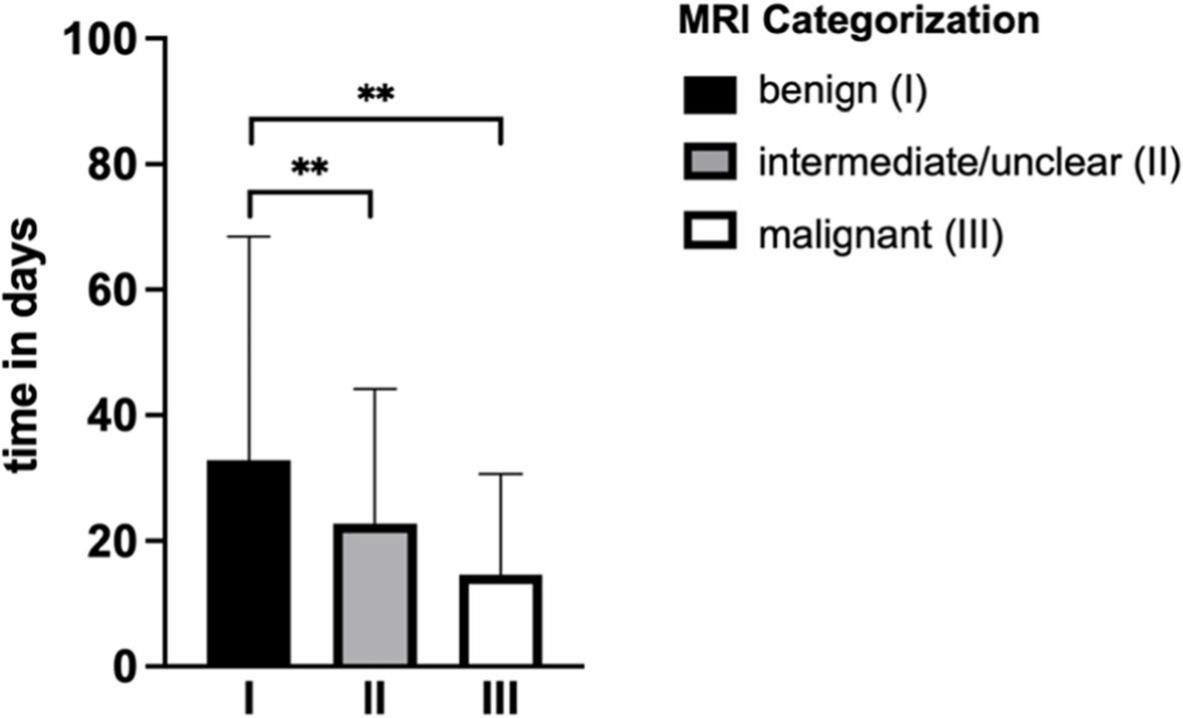
Time interval between the first consultation to UCC and surgery. Statistical significance is indicated by **p < 0.005

Moreover, on average there were 32.8 days (SD: 35.7) between the first contact of the UCC and surgery in patients with suspected benign STT. In comparison, patients with intermediately/unclear classified tumors in MRI-report received surgery after an average of 22.7 days (SD: 21.4). This was significantly shorter compared to benign-rated STT (*p* = 0.008). Treatment was the fastest in patients with suspected malignant tumors in MRI-report. They received surgery after an average of 14.6 days (SD: 16.0). Thus, time delay of consultation of the UCC to first surgery was highly significantly shorter in comparison to patients with STT rated as benign (*p* < 0.0001) (Fig. 5).

**Fig. 6:**
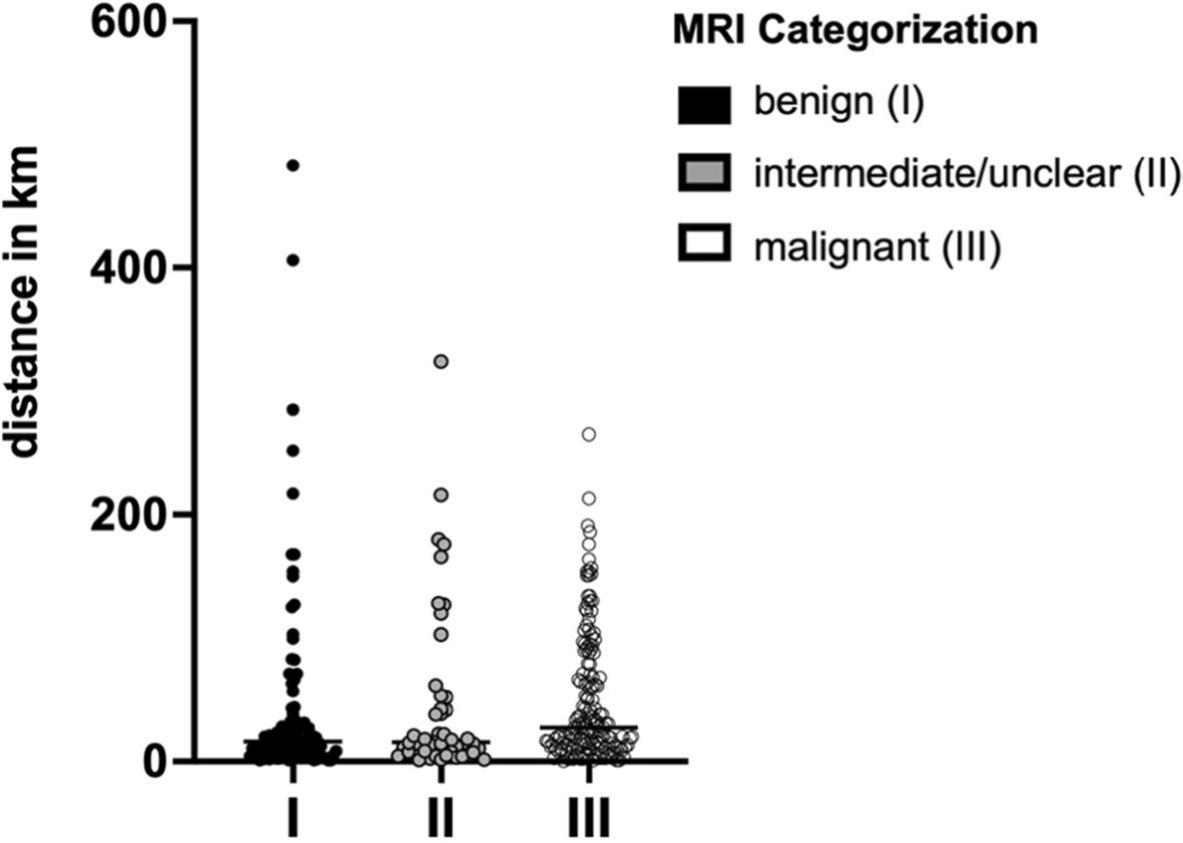
Distances traveled by patients to the UCC. The dot-plot diagram shows the three cohorts distinguished by the degree of suspected tumor malignancy in MRI. A homogenous distribution exists with no statistical difference among the groups

Patients with suspicion of a benign tumor traveled on average 44.1 km (SD:79.1) to the UCC. For the second cohort of patients with intermediate/unclear STT in MRI it was 46.1 km (SD:68.3), and 49.8 km (SD:51.9) for patients with a suspiciously malignant STT in MRI. No statistical differences were observed between the cohorts. Moreover, no correlation was observed between patients´ age and traveled distance (*r* = 0.05692) (Fig. 6).

## Discussion

The presented findings show that the MRI-report and the provisional diagnosis of the lesion influence the treatment speed of patients with soft tissue swellings. This emphasizes the importance of the radiologist´s role. In the MRI report, a direct reference to a sarcoma center or a recommendation for a biopsy should be given in the case of a suspicious mass [6]. In contrast, a delay of further diagnostics (e.g., biopsy) may allow the development of advanced disease [17]. Usually, no direct flow of information between patient and radiologist exists in the case of publicly insured patients (87% of the total population) in Germany [18]. The radiologist sends the MRI report to the physician, who initially referred the patient to MRI diagnostics. Due to data protection laws, communication is often restricted to letters or FAX leading to further time delay. Direct information of the radiologic findings, at least in cases with a suspicion of a malignant STT, via phone call with the recommendation of a specialized center could dramatically reduce the overall diagnostic time delay. Eventually, the patient himself needs to make the appointment at the UCC and consent to an operation. Personal matters of familial, religious, or financial origin might contribute to a further delay in diagnostics. Altogether, these interfering factors contribute to the overall, large standard deviations within the analyzed cohorts.

OnkoZert, the certification program of the German Cancer Society, requests to offer patients an appointment for a biopsy within five working days after detection of a suspicions mass [14]. In our study, the average time between the first contact of the cancer center and surgery was 14.8 days (SD:17.1) for masses suspicious for sarcoma of the soft tissue. It is very difficult to name the main reason for this delay, some aspects are already discussed above. But delays in appointments due to an over-booked outpatient clinic cannot be ruled out. All biopsies were performed as open incision biopsies. Thus, patients underwent standard preoperative procedures. A computed tomography (CT)-guided fine needle aspiration biopsy might be a quicker alternative because no anesthesiologist and surgical team are necessary. However, fine-needle biopsies deliver less accurate results versus open incision biopsies in sarcomas [19, 20].

Younger et al. reported patients with soft tissue and bone sarcomas in England; 48% of the patients saw a doctor within one month of the development of symptoms, 27% within three months, and 31% within a year [2]. Brouns et al. reported similar findings. Here, 80% of patients consulted a general practitioner as their first medical contact with symptoms [15]. Younger et al. also showed that the symptoms are more likely to be misinterpreted by physicians in younger patients than in older patients [2]. Weaver et al. offered two explanations for this: Young patients are more likely to dismiss their symptoms and have a lesser suspicion of cancer in younger patients than older patients by primary healthcare professionals [21, 22].

Our study examined the time between an MRI scan and contact with a specialized UCC. However, the time interval between first recognition of symptoms and contact to a healthcare professional has not been registered, yet. Buvarp Dyrop et al. reported that this time interval is by far the longest when diagnosing sarcomas [16].

Younger and colleagues report that adolescent and young patients are willing to travel further for specialized sarcoma treatment [2]. No correlation of age and traveled distance was found in our cohort. A thinkable reason is the setting of our study, a metropolitan area in Western Europe. In comparison, Younger et al. analyzed data from the whole National Health Service (NHS) England and thus analyzed a complete country in a multi-center study. The average catchment area of the UCC was 47.5 km. Similar institutions can be reached at a distance of 73 km, 92 km, 156 km, and 185 km. Due to the fairly easy accessibility of a specialized center, a selection towards more malignant tumors being treated at the specialized center has not been observed. Moreover, younger patients did not incur longer traveling distances in comparison to older patients. Other authors report worse oncologic outcomes in rural populations versus urban population [23]. Although overall survival has not been analyzed in our study, there is no evidence of discrimination of patients that lived further away from the center, compared to others nearby.

All general limitations of a retrospective analysis apply to this study. Resolution of the MR-tomographs (slice thickness and magnetic field strength in tesla) was not considered. Higher MRI resolution might have led to a more exact diagnosis influence the further treatment of a patient. This major limitation will always exist as long as different MRI scanners are used for patient examination. Patients´ details such as cancer anamnesis, medical professional knowledge, and income level were not considered. All these circumstances can tremendously affect the patient’s disease awareness and seek medical support. This major limitation will be present in prospective and retrospective studies and can hardly be overcome in a multifaceted society. A heterogeneous group of radiologists performed the imaging at multiple institutions. However, decisions on the surgical procedure were performed in one institution by three experienced surgeons, working in the same department. This limitation should have a minor impact on the study since most healthcare providers act by medical guidelines.

## Conclusion

The radiologic report is of utmost importance for the further treatment of soft tissue tumors. The radiologist´s assessment of the tumor can delay or accelerate tumor treatment. In cases of intermediate/unclear or malignant suspicious STT, direct information to the patient by the radiologist with the recommendation of a specialized UCC could significantly speed up further treatment. No age discrimination was observed and slightly more men than women were treated in the studied population. The traveled distance by patients to meet a specialized physician for further treatment of soft tissue tumors did not correlate to the tumor´s degree of suspected malignancy.

## Supplementary Information

**Additional file 1.**

## Data Availability

The datasets generated and analyzed during the current study are available as supplementary material.

## Abbreviations

DDLPS: Dedifferentiated liposarcoma
MRI: Magnetic resonance imaging
MXLPS: Myxoid liposarcoma
NOSLPS: Not-otherwise specified liposarcoma
PMLPS: Pleomorphic liposarcoma
STT: Soft tissue tumor
UCC: University Cancer Center
